# Environmental health aspects of coal ash phytoremediation by selected crops

**Published:** 2011

**Authors:** Jerzy Bilski, Kyle McLean, Erin McLean, Fakira Soumaila, Mardee Lander

**Affiliations:** Valley City State University, Valley City, ND 58072, USA

**Keywords:** Coal ash, phytoremediation, heavy metals accumulation

## Abstract

The objective of this research was to determine the effects of growth media containing FA and FA mixed with soil on selected crop plants seedlings growth. We studied the influence of various FA concentrations (e.g., 0, 10, 20, 30, 40, 50, 60, 70, 80, 90, and 100% of FA in growth media by weight basis) in FA/soil composed media on the germination, growth, and heavy metals uptake of the following plants: barley, Sudan grass, ryegrass, rape, alfalfa, and canola. Plants were grown on Petri dishes (10 cm diameter, 3 replications) for 14–21 days, harvested, dried, and weighed. Experiments have been replicated three times. The concentrations of Al, B, Ba, Co, Cr, Cu, Mo, Sr, Ti, Tl, and V in growth media were determined, and the concentrations of the same elements in young plants were analyzed. Addition of 10, 20, and 30% of FA to the soil were acceptable for most plants, as compared to FA alone used as a growth media. Barley was the only plant of plants used in our research, which was able to sustain seedlings growth on media consisting on FA alone. Preliminary results of chemical analysis of FA and harvested young plants implicate that plants do not accumulate toxic amounts of heavy metals even being grown on media containing 100% FA. Our research results indicate that coal FA might be used as a plant growth media additive. However, additional studies should be undertaken to determine the effects of FA on plants grown till maturity.

## 1. Introduction

Fly ash (FA) is a byproduct from burning pulverized coal in electric power generating plants (Carlson and Adriano, 1993). During combustion, mineral impurities in the coal fuse in suspension and float out of the combustion chamber with the exhaust gases. As the fused material rises, it cools and solidifies into spherical glassy particles called fly ash. Fly ash contains heavy metals and radioactive elements (Patham et al. 2003, Rai et al. 1987).

A vegetative cover is a remedial technique to stabilize coal fly ash (FA) landfills, and to physically and chemically immobilize heavy metals present in FA. (Bilski et al. 1995, Bilski et al. 2011). However, there is a great concern, that plants planted or voluntarily growing on media with high content of FA may absorb toxic amounts of Se and/or heavy metals. Despite these objections, the utilization of FA as a growth medium for plants is an attractive alternative for disposal of FA in landfills (Rethman et al, 2001).

The use of fly ash as an amendment to agricultural soils has been investigated to explore its effects on crop growth and production (Bilski et al. 1995, Siuta 2001, Rosik-Dulewska 2002, Bilski et al. 2011).

Many herbaceous plants, primarily grasses which exhibit rapid growth, are moderately resistant to environmental stress, and are therefore often used as cover crops in environmental restoration and remediation projects (Bilski et al. 1995, Chu 2008). However, there is a great concern, that plants planted or voluntarily growing on media with high content of FA may absorb toxic amounts of Se and/or heavy metals. Despite of these objections, the utilization of FA as a growth media for plants is an attractive alternative for disposal of FA in landfills (Bilski et al 2011).

Coal fly ash, used as soil amendment, or on a soil-covered fly ash landfills, can be very effective as a provider of certain essential nutrients to plants, such as B, Mg, Mo, S, and Zn (El-Mogazi et al. 1988, Gibczynska et al. 2006). However, if the soil is already enriched with a particular element, the addition of more may prove to be toxic to the plant (Openshaw 1992). Fly ash also has been reported to improve the nutritional status of soils by providing plants with micronutrients. But also through the increases in cation exchange capacity (CEC)(Gatima et al. 2005)

We hypothesized that selected plants will grow on media containing FA and/or soil mixed with FA. The objective of this experiment was to study the influence of various FA concentrations in FA/soil composed media on the germination, growth, and uptake of heavy metals by several plant species.

## 2. Materials and Method

Coal FA from Montana semi-bituminous coal alone or in combinations of different soil/FA ratios have been tested as plant growth media for the following plant species: barley *(Hordeum vulgare)*, Sudan grass *(Sorghum bicolor*), canola *(Brasica campestris*), rapeseed *(Brassica napus*), alfalfa *(Medicago sativa*), and perennial ryegrass *(Lolium perenne*).

Adaptation of plants to coal ash based media have been presented with regard to seedlings growth, heavy metals presence in the media and accumulation of heavy metals by young plants.

Plant growth media composition:

The controls of 100% Soil (100Soil), and 100% NDSU FA (100ND)

1. 90% NDSU Ash 10% Soil (90ND 10Soil)
2. 80% NDSU Ash, 20% Soil (80ND 20S)
3. 70% NDSU Ash, 30% Soil (70ND 30S)
4. 60% NDSU ASH, 40% Soil (60ND 40S)
5. 50% NDSU Ash, 50% Soil (50ND 50S)
6. 40% NDSU Ash, 60% Soil (40ND 60S)
7. 30% NDSU Ash, 70% Soil (30ND 70S)
8. 20% NDSU Ash, 80% Soil (20ND 80S)
9. 10% NDSU Ash, 90% Soil (10ND 90S)

### Planting

Plants were grown on Petri dishes (3 replications for each plant and treatment) for 14–21 days, harvested, dried, and weighed. The number of seeds planted per Petri dish depended on seed size.

### Watering: Done each morning

Plant growth media were watered to the level of becoming fully-moist, but without excess standing water (so called “soil paste” watering level).

### Harvesting

After two to three weeks of growing, plants were cut at growth media level.

All plants were measured, the envelopes were labeled, and the plant material was placed inside the envelopes.

Samples were then placed into the oven to dry, and after two weeks the dry plant material was weighed.

### Analyses

The dry and weighed samples were treated through an acid digestion process.

The samples were then put through an inductively coupled plasma (ICP) emission spectrophotometry to determine the presence and concentration of selected elements. The concentrations of Al, As, B, Ba, Be, Cd, Co, Cr, Cu, Mo, Sr, Ti, Tl, and V in growth media have been determined, and the concentrations of the same elements in young plants have be analyzed.

## 3. Results

**Figure 1:**
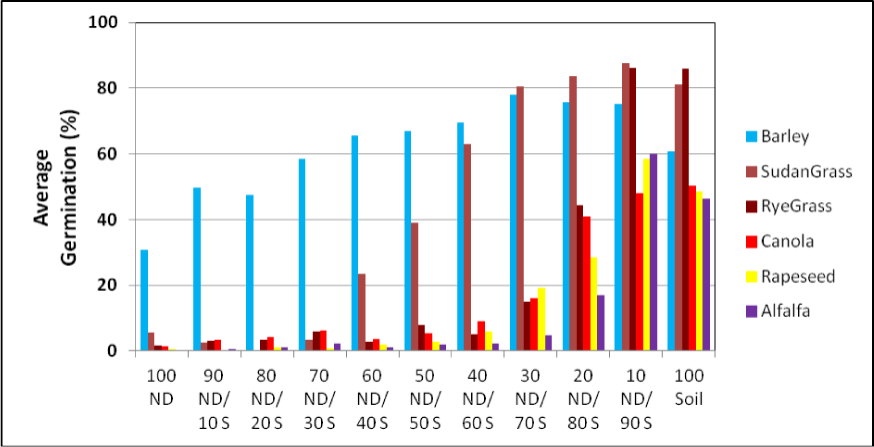
Germination of selected plants in growth media containing different concentration of FA in soil.

**Figure 2:**
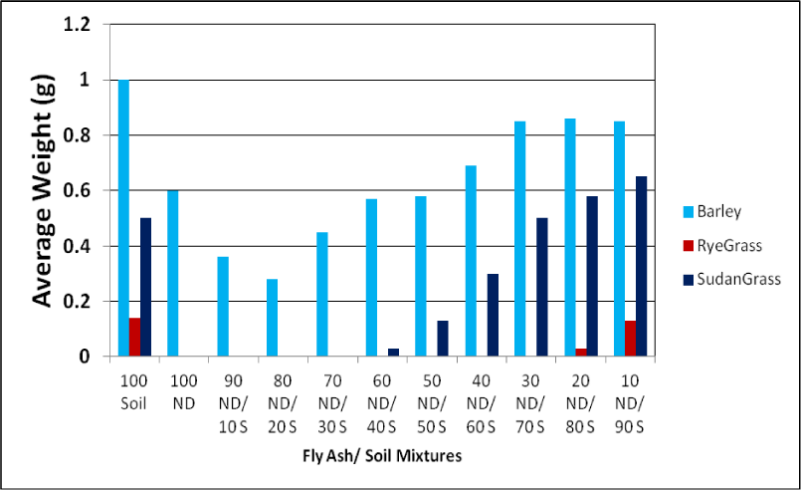
The weight of barley, ryegrass, and Sudan grass seedlings grown on media containing different concentrations of FA and soil

**Figure 3:**
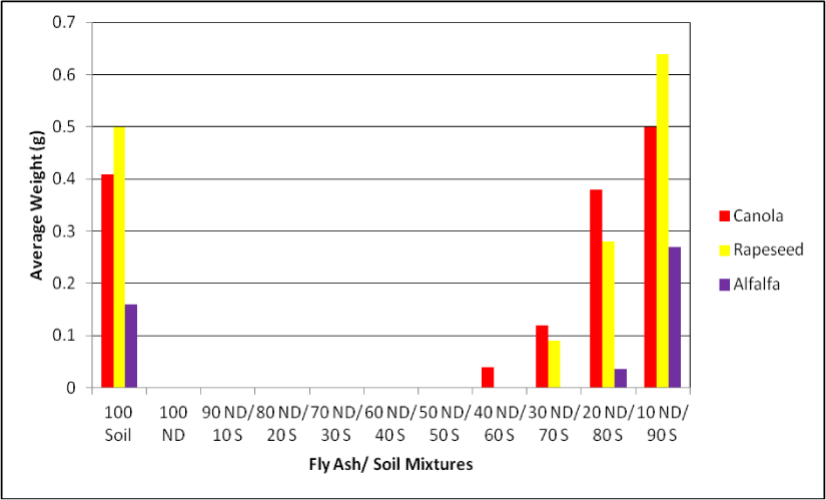
The weight of canola, rapeseed, and alfalfa seedlings, grown on media containing different concentrations of FA and soil.

Effects of growth media containing different concentration of FA in soil on plant germination and weight are presented in Figures 1, 2, and 3.

All plants were able to germinate on growth media containing up to 40% of FA in growth media. Such concentration of FA in the media, despite still supporting germination, reduced the level of germination of all plants significantly, as compared to the control. Barley and Sudan grass expressed the lowest level of this reduction. The concentrations of FA in growth media higher than 40% were not able to sustain growth seedlings after initial germination, except for barley and Sudan grass.

Barley and Sudan grass showed not only the greatest germination but also the highest seedlings weight of all tested species, despite being grown in harsh conditions, with gradually growing concentrations of FA in the media. Barley seems to be the most adaptable to the growth on FA of all plant used in our study. Barley was the only plant of plants used in our research, which was able to sustain seedlings growth on media consisting on FA alone.

Ryegrass and alfalfa had the poorest growth on media containing FA.

### Chemical Analysis

 

## 4. Discussion

The concentration of aluminum (Al) in all plant growth media didn’t exceed the concentrations found in some American soils (Mirasol, 1920; ATSDR, 2006). Despite this, the concentrations of Al in plant tissues in our experiments were very high, reaching almost 2000 mg/kg in rye seedlings grown on 100% FA).

The concentration of arsenic (As) in plants was below the detection limits, despite relatively high concentration of As in FA used as growth media (up to 64 mg/kg). Such concentration meets the standards for considering plant growth media as phytotoxic (Kabata-Pendias and Adriano, 1995).

The concentration of boron (B) in all growth media containing FA reached 1230 mg/kg in FA and exceeded several times values expected to be present in the soil (usually up to 26–33 mg/kg; ATSDR., 2006). Growth media supplemented with FA in our experiments contained significantly higher amounts of B, than noted in literature (Mikkelsen and Camberato, 1995). The highest levels of B concentration in plant tissues reached 468 mg/kg (highest levels in in triticale, rye, and oats seedlings). In addition, plants expressed the most common symptom of excessive B accumulation which is the necrosis along leaf margins and at the growing points, reflecting the buildup of B following the transpiration stream (Mikkelsen and Camberrato, 1995).

Barium (Ba) is found in most soils at concentrations ranging from about 15 to 3,500 mg/kg and mean values ranging between 265 and 835 mg/kg, depending on soil type (ATSDR 2007). Barium in our soil, control treatment reached the level of 1900 mg/kg, and was within acceptable limits for soils. Barium in our FA only slightly exceeded acceptable limits for soils. The concentration of Ba in plant seedlings, although strongly related to the concentration in growth media, remained within toxicologically acceptable limits.

The average concentration of cobalt (Co) in soil in the United States is 7.2 mg/kg, with a range of 1–40 mg/kg (Smith & Carson, 1981). The concentration of Co in the soil used in our experiments was close to listed above average, but in media containing FA significantly exceeded the amount found in the soil. Despite elevated amounts of Co in our growth media, there is little evidence of Co toxicity to plants due to elevated concentrations in soil or other growth media (USEPA 2005).

In most soils, chromium (Cr) occurs in low concentrations (2 – 60 mg/kg) and in our studies not only Cr concentration in the soil, but also in all but one FA based plant growth media Cr concentration remained within these limits (Kabata-Pendias and Adriano, 1995).

Although plant growth media supplemented with FA contained elevated amounts of copper (Cu), as compared to the soil, it didn’t result in elevated concentration of Cu in plant tissues. The concentration of Cu remained almost unchanged and within average values for Cu concentration in plants (INCHEM, 1998).

High concentration of molybdenum (Mo) is generally associated with alkaline soils. Molybdenum concentration up to 50 mg/kg dry weight has been found in agricultural soils (Hornick et al., 1977). Although soil pH was alkaline (pH 8.3–8.5) we didn’t notice elevated amounts of Mo in soil used in our study. Concentration of Mo in FA media was a few times higher than in the soil, but didn’t reach mentioned above extreme values. Despite this, plants grown on media with elevated concentration of Mo showed significantly elevated accumulation of Mo, as compared to plants grown on the soil. Concentrations of Mo in plant s above 10 mg/kg might be dangerous for animals fed with these plants (Kabata-Pendias and Mukherie, 2010), but in our studies plants grown on Mo rich media didn’t exceed this concentration.

Strontium (Sr) is found naturally in soil in amounts that vary over a wide range, but the typical concentration is 250 mg/kg. The concentration of Sr in FA may vary depending on FA source and be as low as < 1mg/kg and as high as 3,900 mg/kg (Mattigod et al., 1999). In our experiment, the concentration of Sr in the soil was 250mg/kg, and the concentration in FA was higher than typical concentration for FA, reaching 4300 mg/kg. Plant accumulation of Sr was correlated to the high concentration in FA based media, and for all plants grown on FA ranged between 300 to 500 mg/kg. Despite such high accumulation of Sr in plants, it does not seem to create any toxic effects, because frequently Sr levels in forage crops reach even higher values than in our experiments (Kabata-Pendias and Adriano, 1995).

Heavy clay soils, like the soil used in our experiment (Fargo clay) contain more titanium (Ti) than sandy soils (Monier-Williams, 1950), and the average concentration in soil appears to be below 5 g/kg. Our soil contained only a fraction (about 10%) of this amount, and our FA didn’t contain less than 5 g Ti per kg. The concentration of Ti in plant s was also within acceptable limits (Kabata-Pendias and Mukherie, 2010).

The vanadium (V) content if soils is related to those of the parent rocks from which they are formed and range from 3 to 310 mg/kg (Waters, 1977). The concentration of V in all our growth media didn’t exceed 100 mg/kg, and as a consequence, concentrations in plant tissues remained low.

The concentration of As, Be, Cd, Pb, and Tl in plants were below ICP detection limits.

## 5. Conclusions

1. The additions of 10, 20, and 30 % of FA to the soil supported plant germination and growth.
2. Preliminary results of chemical analysis of FA and harvested young plants implicate that plants do not accumulate toxic amounts of heavy metals, even grown on media containing 100% FA.
3. These results indicate that coal FA might be used as a plant growth media supplement.
4. Additional studies should be undertaken to better understand the effects of FA on plant growth from germination to maturity.
5. Barley and Sudan grass expressed the highest phyto-remediatory potential for coal FA.
6. Using our studies, application of different plant species for the revegetation of coal ash piles might be discussed.

## Figures and Tables

**Table 1:** Concentration of trace elements in soil, FA, and plants

	Al	B (mg/kg)	Ba	Co (mg/kg)	Cr (mg/kg)	Cu (mg/kg)	Mo (mg/kg)	Sr (mg/kg)	Ti	V (mg/kg)
Soil	1.43%	19	0.19%	6.81	17.3	15.8	2.9	200	0.05%	27.3
FA	6.28%	1230	0.35%	12.29	39.6	32.9	10.3	4300	0.14%	83.8
Highest concentration in plants	1991 mg/kg	468	221 mg/kg	1.99	4.41	11.7	7.9	500	135.5 mg/kg	3.95
